# A Comparison of Strategies for Recruiting Teachers Into Survey Panels

**DOI:** 10.1177/2158244018796412

**Published:** 2018-08-22

**Authors:** Michael W. Robbins, Geoffrey Grimm, Brian Stecher, V. Darleen Opfer

**Affiliations:** 1 RAND Corporation, Pittsburgh, PA, USA; 2 RAND Corporation, Arlington, VA, USA; 3 RAND Corporation, Santa Monica, CA, USA

**Keywords:** educational research, survey methods, response rates, incentives, sampling bias, survey panels

## Abstract

We examine a range of options for recruiting teachers into a nationally representative survey panel. Recruitment strategies considered include a telephone-based approach and the use of promised incentives and pre-incentives of varying amounts and forms. Using a randomized experiment, we evaluate the effectiveness of five separate recruitment strategies and conduct a cost-benefit analysis. Our preferred strategy is one that uses a US$10 gift card as pre-incentive (it yielded a 27% rate of successful recruitment a cost of US$78 per recruited teacher). Statistical comparisons indicate that no other technique was superior to this strategy in terms of recruitment rate or cost-effectiveness. Efforts at refusal conversion after the initial approach were mostly ineffective. A comparison across demographic type characteristics of enrolled panelists and nonrespondents shows no substantial differences for any recruitment strategy considered. Hence, the potential for recruitment-level nonresponse to induce large bias into findings from surveys administered to the panel is minimal.

## Introduction

Recent U.S. education policy is designed to promote large-scale, long-term improvements in the public education system—more challenging curriculum standards, more effective teachers, deeper learning, richer assessments, improved college, and career readiness. Changes of such magnitude require individual educators to learn new information, modify expectations, and master new behaviors—changes that take time to occur. Yet, the tools we have for monitoring the education system are static—they take the pulse of the system at one point in time but do not link data across time. Consequently, we cannot monitor the implementation of these reforms in ways that would help us track changes, identify problems, and craft solutions. Policy makers and the public would be better equipped to deal with anticipated changing policies if they were able to track the uptake of such policies, the responses of school leaders, and the evolving consequences for teachers, both anticipated and unanticipated.

Although national surveys periodically provide valid cross-sectional data on teachers or school leaders, policy makers and the public seldom have access to valid longitudinal data. Having longitudinal information “creates an unfolding story”^1^ of the effects of reforms on educators, and this ultimately helps us understand how these reforms are likely to affect student outcomes. A better understanding of the evolving story would help policy makers and practitioners take steps to meet the changing needs of educators as they implement new policies—for example, new standards, curricula, assessments, and teacher and principal evaluation systems. To address this need, we attempted to recruit a representative teacher panel to respond to surveys about changing education policies three to four times per year over an extended period of time. The purpose of this panel, which is RAND’s American Teacher Panel (ATP), was to provide a rich source of information on teachers’ evolving knowledge, attitudes, practices, and the conditions in which they work.

This article concerns nonresponse (including nonresponse biases) at the recruitment phase for the ATP and details efforts that were made to improve the rate of successful recruitment and perhaps reduce nonresponse biases related to recruitment. The research on strategies for panel recruitment, which is an issue that is distinct from that of survey response, is notably sparse despite the fact that low rates of recruitment into survey panels can be just as detrimental to the validity of analyses as low rates of response to specific surveys. Hence, our literature review mainly focuses on issues pertaining to nonresponse on cross-sectional surveys.

The primary concerns with regard to the reliability of survey findings are the accuracy (i.e., lack of bias) and precision (i.e., low variability) of the survey estimators. Nonresponse on surveys can be detrimental to both the accuracy and the precision of the survey findings. Respondents may be inherently different from nonrespondents, which induces the potential for biased findings. (See Edwards et al., 2002; Singer, 2002; and Singer and Ye, 2013 for thorough reviews of the relevant literature.) The potential for the presence of nonresponse bias may be assessed by comparing the breakdown of respondents and nonrespondents across characteristics that are observed for all individuals who were asked to take the survey (e.g., demographics). It is assumed that high rates of response help mitigate biases; however, meta-analyses have shown that high rates of nonresponse do not necessarily indicate the presence of substantial nonresponse bias nor do low rates of nonresponse assure a lack of bias (Groves & Peytcheva, 2008; Leslie, 1972). Regardless, higher response rates do yield a larger (and therefore more precise) sample. For these reasons, there is a vast literature on survey response rates.

Nearly every factor that might conceivably influence response rates has been studied to some degree—questionnaire design (Dillman, Sinclair, & Clark, 1993), confidentiality statements (Dillman, Singer, Clark, & Treat, 1996), personalization of an outside envelope (Kahle & Sales, 1978) or of a letter’s salutation (Heerwegh, 2005), advance letters (Mann, 2005; Parsons & Medford, 1972), and deadlines for completion (Roberts, McCrory, & Forthofer, 1978). For an in-depth review of all facets of survey administration, see Dillman, Smyth, & Christian, 2014. Despite the multitude of minutiae that may influence responsiveness, our focus here is on mode of contact and incentives for participation.

As technology has improved over the years, the ways in which a potential survey respondent can be contacted (e.g., face-to-face, mail, telephone, email) has increased dramatically—response rates and data quality can vary substantially depending upon the mode used (see Dillman et al., 2009; Kaplowitz, Hadlock, & Levine, 2004; Porter & Whitcomb, 2003; Schaefer & Dillman, 1998). More costly modes of contact such as mail and phone tend to yield higher rates of response than less expensive options such as email. Researchers commonly vary the mode across the initial contact and follow-up.

Survey methodologists have developed a myriad of ways to incentivize sampled individuals into responding to a survey; the types of survey incentives that have been studied range from promised payments to charity (Hubbard & Little, 1988) to enrollment of the respondent in a lottery (Warriner, Goyder, Gjertsen, Hohner, & McSpurren, 1996). However, we focus on incentives that involve payment to the respondent delivered upon completion of the survey (i.e., a promised, or contingent, incentive; Berry & Kanouse, 1987) and payment to the respondent delivered upon receipt of the survey (i.e., a pre-incentive or noncontingent incentive; Armstrong, 1975). The consensus in the literature is that pre-incentives are more effective at increasing response rates than all other forms of incentive, whereas promised incentives do not always increase response over strategies that offer no incentive (Church, 1993; Edwards et al., 2002). In addition, several researchers have searched for an optimal magnitude of pre-incentive (Dykema et al., 2015; James & Bolstein, 1990; Mizes, Fleece, & Roos, 1984; Trussell & Lavrakas, 2004). Generally, the observed improvement in responsiveness in surveys increases with increasing incentive value; however, a leveling off of the effect is often reported (Godwin, 1979; James & Bolstein, 1992). Singer and Ye (2013) cite numerous studies that fail to find an effect of incentives on item nonresponse and other measures of response quality. Other authors have shown that incentives also can be useful as a means of refusal conversion (see, for example, Kropf & Blair, 2005 and Zagorsky & Rhoton, 2008; note that the latter reference considers refusal conversion within panel surveys).

Only a few of these studies include a cost-benefit analysis across various modes of contact and/or incentives (e.g., Martin, Duncan, Powers, & Sawyer, 1989; Newby, Watson, & Woodliff, 2003; Shaw, Beebe, Jensen, & Adlis, 2001; Teisl, Roe, & Vayda, 2006). Knowing that one mode of contact or incentive is more effective than another in encouraging respondents to complete a survey is only part of the information needed to decide which approach will yield the best overall outcome when there are constraints on funds for fielding surveys (the normal situation). It is necessary to know the relative costliness of the type of contact, the size of the incentives, and the relative effectiveness in terms of response rates. Most studies that include cost-benefit analyses report that larger incentives are not cost-effective in comparison with smaller incentives, although expensive modes of contact are often cost-effective. No study that we have seen that investigates cost-effectiveness uses statistical inference to compare strategies.

In addition, few studies of survey response rates and non-response bias focus specifically on teachers, although low response rates are common among this group (which may be due in part to teachers facing a higher survey burden than other professionals); response rates of 20% to 30% when pre-incentives are not used have been reported by multiple authors (e.g., Fraze, Hardin, Brashears, Haygood, & Smith, 2003; Dykema, Stevenson, Klein, Kim, & Day, 2013). Other groups of professionals have been studied, such as school principals (Jacob & Jacob, 2012), physicians (Grava-Gubins & Scott, 2008; VanGeest, Johnson, & Welch, 2007), and college faculty (Schuldt & Totten, 1994; Weible & Wallace, 1998); these groups also tend to yield poor rates of response.

Finally, we summarize existing literature on recruitment for survey panels, which less is prominent than the literature on cross-sectional surveys (although survey panels have gained in popularity in recent years). Göritz (2004) studies recruitment for online access panels but does not consider monetary incentives. There is research to suggest that pre-incentives increase responsiveness of panel participants to specific surveys (Creighton, King, & Martin, 2007; Goldenberg, McGrath, & Tan, 2009). Furthermore, Singer and Kulka (2002) and Creighton et al. (2007) observe that it is not necessary to use incentives in each wave of a longitudinal survey to maintain desired rates of response. Scherpenzeel and Toepoel (2012) study the effect of incentives and mode in recruitment for an online panel in the Netherlands; they find, among other things, that prepaid incentives clearly outperform promised ones. A thorough overview of the current state of the literature on online panels is found in Callegaro et al. (2014).

The article proceeds as follows. The recruitment methods, including the experiment and specific strategies, are detailed in the next two sections. Then, we provide a thorough analysis that explores nonresponse bias (i.e., we compare enrolled panelists to nonresponding teachers across a wide range of observable characteristics), and we briefly consider whether panel members display differential response rates (on the basis of the strategy through which they were recruited) on surveys administered subsequently to the panels.

## Method

The present study was conducted as part of a larger effort to recruit teachers into the ATP. Here, we detail the steps of recruitment of these educators for the purpose of comparing recruitment strategies.

The first step in recruitment for the panel was to draw a random sample of 1,572 U.S. public schools. There was moderate oversampling of larger schools. Rosters of the schools’ active teachers were purchased from a vendor. A variable number of teachers at each school were selected from the school’s teacher list and asked to enroll in the ATP. The number of teachers chosen from each school varied depending upon the size of the school; on average, four teachers were selected from each school (in all, 6,590 teachers were selected at this stage). A successful recruitment is when a contacted teacher elects to enroll in the panel by filling out a provided form that contains basic information; teachers who decline to enroll are nonrespondents.

These initial 6,590 teachers are referred to as Group 1 throughout this article. The approach for contacting them, which was designed to be comparatively inexpensive, used a promised incentive, and elaboration is provided below. Unfortunately, recruitment within Group 1 produced notably low rates of enrollment (approximately 11% of teachers contacted agreed to join the ATP). This enrollment rate left the panel well short of its targeted number of participants. Furthermore, this phase of recruiting yielded a relatively high cost per recruit (approximately US$70 per teacher recruited).

Additional recruitment was needed to bolster the size of the ATP, and we decided to conduct an experiment to compare a variety of recruitment strategies using different forms of incentives. Specifically, a group of 1,000 previously uncontacted teachers was divided into four randomly selected subgroups (each of size 250, which was determined to be large enough to detect meaningful discrepancies between groups), and a different strategy for recruitment was applied to each subgroup—the experimental group of 1,000 teachers is referred to as Group 2, and the subgroups, each of which received a different recruitment strategy, are referred to as arms of the experiment. Briefly, the four arms involved different incentives: US$10 and US$20 (physical) gift cards as pre-incentive, US$20 electronic gift cards as pre-incentive, and phone-based recruitment with promised incentive, respectively.

In addition, we also investigated the feasibility of improving recruitment rates among nonrespondents from the Group 1. Specifically, as a means of refusal conversion, the most successful of the experimental strategies (which proved to be a US$10 pre-incentive) was applied to a randomly selected subgroup of teachers from Group 1 who were contacted but did not respond. The subgroup to which refusal conversion was applied is referred to as Group 1a.

To summarize, our study was designed to address three specific research questions:

**Research Question 1: Which strategy for recruitment of educators into survey panels is most effective?**
**Research Question 2:** Which strategy is most cost-effective in terms of cost per recruited educator?
**Research Question 3:** Are any of the strategies effective (in terms of recruitment rates and costs) at converting refusing educators?

**Figure 1:**
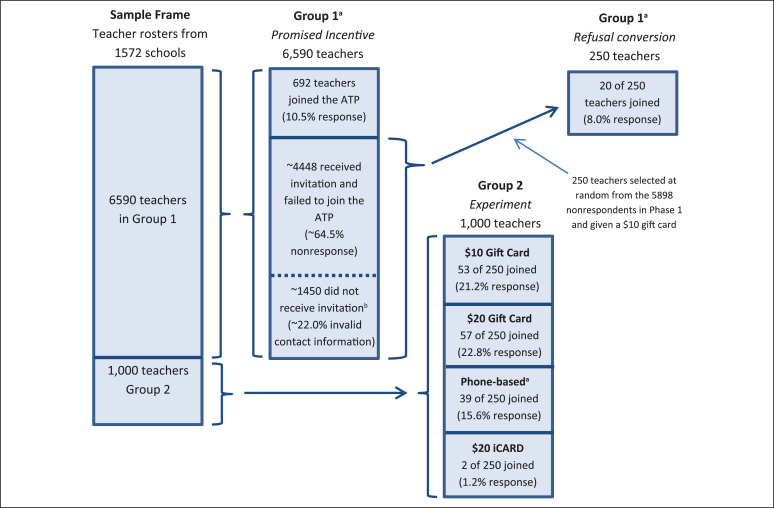
Flowchart illustrating the study design for recruitment of teachers.

*Note*. ATP = American Teacher Panel.

^a^Group 1 and the phone-based arm of Group 2 used a promised incentive. All other recruitment efforts involved a pre-incentive.

^b^It is believed that a portion of the contacted teachers in all groups did not receive the invitation to join the ATP due to invalid contact information (although this nuance is only illustrated for Group 1 in the figure). The portion of teachers with invalid contact information is estimated from the phone-based arm of the experiment and is extrapolated to the other arms and groups for subsequent analyses.

The various phases of this study were administered from December 2014 to February 2015. A flowchart illustrating the study design as applied to teachers is shown in Figure 1. Note that the chart illustrates findings (e.g., response patterns) and other nuances (e.g., uncontacted educators) that are described in the subsequent sections.

We describe all components of each recruitment strategy considered here, as well as the cost incurred by the components, in detail in the ensuing text—to assist the reader, this information is also rendered within in Table 1.

**Table 1:** The Components Used Within the Recruitment Strategies and the Cost Incurred From Each Component.

	Recruitment strategy component	Group 1	Group 1a^a^	Group 2: experimental comparisons				Cost^b^
				US$10 gift card	US$20 gift card	US$20 iCard	2D phone	
	FedEx mailing	✓	✓	✓	✓		✓	US$4.78
Items included in FedEx package	Cover letter^c^	✓	✓	✓	✓		✓	US$0.10
	Brochure	✓	✓	✓	✓		✓	US$0.71
	Noncontingent US$10 Target gift card		✓	✓				US$10.00
	Noncontingent US$20 Target gift card				✓			US$20.00
Items sent electronically	Contingent US$10 iCard	✓					✓	US$10.00
	Noncontingent US$20 iCard					✓		US$20.00
	Opening email^c^	✓	✓	✓	✓	✓	✓	US$0.00
Follow-up with nonresponders	First follow-up email^c^	✓	✓	✓	✓	✓	✓	US$0.00
	Second follow-up email^c^	✓	✓	✓	✓	✓	✓	US$0.00
	Phone calling							US$22.15
	Subsample size	6,590	250	250	250	250	250	

*Note.* ATP = American Teacher Panel.

a Group 1a involves application of all conditions in Group 2a to 250 randomly selected nonrespondents from Group 1 (nonrespondents are those who had not enrolled at the conclusion of all Group 1 email follow-up). Note that this involves repeating certain items (e.g., cover letter, brochure, emails) to individuals in Group 1a.

b Only the cost of the contingent US$10 Target gift card is incurred on a per recruit basis—all other costs are incurred on a per contact basis.

c Item mentions the use of incentives while in the ATP.

### Group 1 (Promised Incentive)

For Group 1 (6,590 teachers), a four-step recruitment strategy was used to recruit teachers. First, a recruitment email was sent to the teachers notifying them of the study and their opportunity to participate; the email also included a link to an online registration page and personal login pin. A follow-up email was sent about a week later. At the same time, we also sent each potential panel member an overnight mail envelope that contained a copy of the recruitment letter, a brochure, and enrollment instructions. A final email was sent to coincide with, or come a day after, the letter was received by the teacher. At each step of contact, the teacher was notified that a promised incentive of a US$10 iCard gift card that would be delivered electronically should he or she agree to participate in the ATP. iCard is an electronic gift card that can be redeemed online for a gift (in this case of US$20) at dozens of well-known retailers. Respondents were also told that once they became panel members, they would be invited to respond to surveys and receive cash incentives based on the length of the survey (e.g., US$25 for a 30-min survey).

### Group 2 (Experimental Comparison of Strategies)

An experiment was used to compare other strategies for recruitment of teachers. We randomly selected 1,000 schools from the list of schools from which teachers were sampled as described above. These schools were then randomly divided into four groups of 250; these define the four experimental subgroups. Next, one teacher from each school who had not been contacted during the first phase was randomly selected to be recruited into the ATP. The four strategies (one of which was applied to each subgroup of teachers) are as follows.

*US$10 gift card as pre-incentive*. Each of the 250 teachers in the first arm of the experiment was sent via FedEx the revised recruitment letter, a brochure on the ATP, and a US$10 Target gift card.

*US$20 gift card as pre-incentive*. Teachers in the second arm of the experiment were administered a larger pre-incentive to examine differences in response rates related to incentive size. The US$20 pre-incentive group received the same recruitment letter and brochure as the US$10 group, but with a US$20 Target gift card.

*US$20 electronic gift card (iCard) as pre-incentive*. The third experimental arm received a US$20 iCard electronic gift card pre-incentive and all recruitment letters via email. Respondents had to click on a unique, secure link in their email and then select a retailer. They would then receive a US$20 electronic gift card for that retailer. The email recruitment letter was altered slightly from the letter received by the other groups to accommodate the iCard link and information.

*Telephone contact with US$10 promised incentive*. A phone-based recruitment strategy with a promised incentive (a US$10 iCard) rather than a pre-incentive was applied to teachers in the fourth experimental arm. This strategy is identical to the strategy applied to Group 1 of recruitment (i.e., recruitment letters sent by email and FedEx) with a single exception: Trained recruiters began calling teachers within this group 2 weeks following the initial email contact. Each of the 250 teachers was contacted until recruitment, refusal, or a maximum of five attempts was reached.

Note that same cover letter was included in the recruitment package for all recruitment strategies; however, one paragraph in this letter was modified as needed to describe the incentive used (when an incentive is used).

### Group 1a (Refusal Conversion)

Finally, the efficacy of these strategies as a means of refusal conversion was evaluated. For our purposes, a refusing teacher (or nonrespondent) is one who is recruited but does not join the panel for any reason. Nonrespondents may include active refusals (in that the teacher consciously decides that they do not want to participate in the panel), passive refusals (in that the teacher intends to enroll but becomes distracted), teachers for whom we have invalid contact information, and so forth.

Refusal conversion was applied to 250 randomly selected nonresponding teachers from the first recruitment group—we refer to these 250 teachers as Group 1a. Specifically, the US$10 gift card pre-incentive strategy (our preferred strategy) was applied to Group 1a. Note that refusal conversion was not applied to all refusing educators. Therefore, the recruitment rates observed during the refusal conversion step are extrapolated to provide estimations of the percentage of educators (out of all of those contacted from Group 1) that would have agreed to join the ATP had the refusal conversion efforts been applied exhaustively; this yields a cumulative response rate.

### Relative Cost

We also wished to perform a cost-benefit analysis that would compare the cost per recruited educator under each recruitment strategy we used. The costs incurred by each component of each recruitment method are listed in Table 1. These cost sources included mailing recruitment letters (including the cost of sending a letter via FedEx, the cost of printing a brochure, and the labor required to prepare the envelopes), pre- and promised incentives, and the labor required to do telephone contacts. There was also a cost of US$0.45 per name to purchase contact information for sampling (i.e., the list of teachers purchased from a vendor)—as this cost was incurred for all strategies, it is not listed in Table 1. All costs, with the exception of promised (i.e., contingent) incentives, are listed as dollar amounts incurred for each educator contacted; for promised incentives, costs are incurred on the basis of each educator who agrees to participate in the ATP (i.e., per recruit).

Our analyses excluded other costs such as the cost of obtaining a nationally representative sample of schools, the fixed cost required to encode the demographic data collection in a web portal, and costs for researcher time (e.g., time spent designing the survey instrument and recruitment tactics, time spent compiling and analyzing findings, etc.)—these costs can be harder to quantify and are mostly independent of the specific recruitment strategy employed (i.e., these costs do not influence comparative cost-effectiveness of the various strategies).

### Unreached Educators

There was one other factor that influenced our estimation of the recruitment rate and the cost of recruitment—the sample members who were no longer working as teachers or who were ineligible for other reasons. For example, a portion of teachers targeted for recruitment did not receive the invitation to participate in the panel due to invalid contact information. We learned about these sampling errors from email bounce backs, overnight mail return notifications, and responses to phone calls that indicated a teacher had left employment or moved schools. However, our ability to discern whether an invitation was not received is highly dependent upon the mode of contact used (and therefore, the recruitment strategy employed).

Unfortunately, each of these methods of determining when invalid contact information was used is imperfect. For example, overnight mail returns are an unreliable indicator of whether or not our attempts to contact an educator were successful as there are several reasons why the information that an educator is no longer at a listed address may not be relayed to the sender. For instance, the delivery person may not distinguish between a refusal and circumstances where an addressee is not at the listed school. Likewise, a school’s front office may accept a package prior to realizing that the addressee is not at the school.

Email bounce backs are also a poor indicator of prevalence of invalid contact information for several reasons. First, an email account may remain active even if it is no longer its user’s primary work address and is therefore not regularly monitored. Second, a teacher may change schools while maintaining the same email address, in which case, their postal address is invalid. In addition, email bounce backs could be due to district spam filters rejecting recruitment emails and unrelated to whether a teacher is at the school.

The phone-based recruitment strategy provides the most accurate representation of the percentage of our sample with invalid contact information. With phone contact, each case involved verbal communication between representatives of the ATP (i.e., trained recruiters) and the respective school; therefore, we were able to quickly receive confirmation of whether the teacher in question was at the school. It is possible that in some cases, our phone recruiters were told a teacher was not at the school as a way to deter further calls. In the phone-based arm of the experiment, 22% (with a standard error of 2.6%) of the targeted teachers were found to no longer be at the school or have invalid contact information. Therefore, we believe it is fair to assume that the overall portion of teachers targeted across all phases of recruitment who did not receive the invitation is approximately 22%. We use the term “effective response rate” to refer to the rate of response among the 78% of teachers who are presumed to have received the invitation.

## Results

### Response Rates

Estimated raw and effective rates of successful recruitment of teachers (along with standard errors) for all recruitment methods are provided in Table 2. The table also provides an estimate of the cost per recruited educator (with standard error) for each phase/strategy. Furthermore, Table 2 provides a cumulative recruitment rate (and associated cost) estimated across Group 1 and the refusal conversion effort (Group 1a)—this rate of recruitment is estimated by extrapolating the success rate of the third phase to all 6,340 nonresponding teachers from the first phase.

**Table 2:** Results for Recruitment of Teachers.

	Group 1	Group 2 (experimental comparisons)				Group 1a (refusal conversion)	
	Promised incentive	US$10 gift card	US$20 gift card	US$20 iCard^d^	Phone-based	US$10 gift card (Group la only)	Cumulative
Educators contacted	6,590	250	250	250	250	250	—
Raw rate of Recruitment							
Estimate	10.5%	21.2%	22.8%	1.2%	15.6%	8.0%	17.9%
*SE*^c^	0.4%	2.6%	2.7%	0.7%	2.3%	1.7%	1.5%
*p* value^a^	000	—	666	000	106	000	274
Effective rate^b^							
Estimate	13.5%	27.2%	29.2%	1.5%	20.0%	10.3%	23.0%
*SE*	0.6%	3.7%	3.5%	0.8%	3.0%	2.2%	2.1%
Cost per recruit							
Estimate	US$69.93	US$77.92	US$116.32	US$1,626.24	US$191.54	US$200.88	US$121.30
*SE*	US$2.11	US$9.50	US$13.54	US$978.03	US$26.71	US$43.08	US$10.51
*p* value	.411	—	.020	.096	.000	.005	.002

*Note.* Strategies in bold use a pre-incentive; otherwise, a promised incentive is used.

^a^p values provide comparisons to the strategy that offers a US$10 gift card as a pre-incentive.

^b^The effective rate is the estimated rate of recruitment among the presumed 78% of contacted teachers who received the invitation.

^c^SEs and p values for raw recruitment rates (noncumulative) are calculated using the normal approximation to the binomial. Effective recruitment rates and costs per recruit are calculated using the (multivariate) delta method. For example, if the raw recruitment rate for a strategy is $\overset{⌣}{p}$ and if the strategy costs US$*A* per educator contacted (with no costs incurred on a per recruit basis), the estimated cost (in US$) per recruit is given by $A/\overset{⌣}{p}$ and the SE of the estimated cost is given by $A\sqrt{(1-\overset{⌣}{p})/\left(n{\overset{⌣}{p}}^{3}\right)}$. SEs and p values for all cumulative rates (and costs) are calculated using the multivariate delta method.

^d^The normal approximation does not hold for the email group as an insufficient number of successes were observed. Therefore, SEs and p values for this group may be inaccurate.

As noted earlier, the effort to recruit teachers in Group 1 (promised incentive) performed relatively poorly (10.5% successful recruitment). With regard to the strategies implemented within the experiment, the US$20 pre-incentive resulted in the highest effective recruitment rate (29.2%). In contrast, the US$10 promised incentive used with Group 1 yielded the lowest cost per recruit (US$69.93/recruit). Our preferred strategy, however, is the US$10 pre-incentive—this approach yields the best balance of recruitment rate and cost (we observe a 27.2% effective rate of recruitment at a cost of US$77.92 per recruit for this strategy). The phoning and iCard strategies provide no discernible benefits. These strategies yield lower recruitment rates while being more expensive. In fact, the electronic gift card produced an effective recruitment rate of only 1.5%. The table also indicates that these methods are also not effective for refusal conversion. Specifically, the cumulative recruitment rate across the first phase (US$10 promised incentive) and third phase (US$10 pre-incentive to convert refusals) is estimated to be lower (17.9%) than our preferred approach (that offers a US$10 pre-incentive upon initial contact). This observation suggests that the recruitment efforts attempted on Group 1 (without pre-incentive) were not only wasteful but perhaps detrimental to the final recruitment rate.

It is desirable to determine whether or not the differences in rates of recruitment and costs per recruit across the various strategies are statistically significant. Thus, for each strategy and type of estimator (i.e., recruitment rate or cost per recruit), Table 2 gives a *p* value for a two-sided test of a null hypothesis that the estimated parameter (either a raw recruitment rate or a cost per recruit) is equal to the corresponding value for our preferred strategy (the US$10 pre-incentive). We do not report *p* values for the effective recruitment rate as they are identical to the *p* values seen when comparing raw recruitment rates. The hypothesis tests justify our choice of the US$10 pre-incentive as the preferred strategy. Specifically, there is no sufficient evidence to conclude that any recruitment strategy produces a higher rate of success than the US$10 pre-incentive strategy (comparison of recruitment rates between this approach and the US$20 gift card strategy yields a *p* value of .666); nor is there sufficient evidence to conclude that any strategy has a lower cost per recruited panel member than our preferred approach (comparison of costs for the US$10 pre-incentive and promised incentive strategies has a *p* value of .411). In fact, most strategies are clearly less successful and more costly than the US$10 pre-incentive method. Furthermore, we see that the cumulative recruitment rate across the first and third phases is not significantly less than the rate yielded by our preferred strategy (*p* = .274); however, the use of refusal conversion is clearly more costly.

### Nonresponse Bias

As the high rates of nonresponse observed among the various phases of recruitment have the potential to jeopardize the generalizability of findings from surveys that use the ATP, we are interested in studying nonresponse bias. The meta-analysis of Groves and Peytcheva (2008) concludes that nonresponse rates are a poor predictor of nonresponse bias, so the low response rates that we observe are not in themselves indicative of substantial nonresponse bias. Nonetheless, we present diagnostics here that evaluate the potential for bias that stems from nonresponse at the recruitment phase. Specifically, we compare observable demographic type characteristics of panel members to corresponding characteristics of nonrespondents (where nonrespondents include any recruited educator that does not enroll in the ATP). These analyses are repeated for all recruitment strategies considered except for the iCard strategy as this strategy did not successfully recruit enough panel members. We examine individual level characteristics including subject taught and gender. The remaining characteristics are descriptors of the teacher’s school. Other standard demographic information (e.g., race, age) is unknown for responders and/or nonresponders and is thus not considered. All characteristics are categorical—we report the percent of panel members that fall into each category (and likewise for nonresponders). These findings are illustrated in Table 3. We compare categorical frequencies for responders and nonresponders (instead of examining response rates within the various domains) as doing so allows comparisons across strategies that observe differing rates of response.

**Table 3:** Categorical Frequencies Across Several Individual Level and School-Level Domains for Empaneled Teachers (Yes) and Nonresponding Teachers (No).

		Group 1		Group 2						Group 1a	
		Promised incentive		US$10 gift card		US$20 gift card		Phone-based		US$10 gift card	
Variable	Category	No (%)	Yes (%)	No (%)	Yes (%)	No (%)	Yes (%)	No (%)	Yes (%)	No (%)	Yes (%)
Subject	English language arts/social studies	14.9	17.6	10.0	17.0	12.8	10.9	13.8	13.5	11.1	16.7
	General elementary	37.5	38.4	46.1	52.8	39.4	40.0	46.9	54.1	48.6	33.3
	Math/science	11.3	12.1	10.0	1.9	12.2	14.5	8.7	2.7	8.3	22.2
	Other	36.3	31.9	33.9	28.3	35.6	34.5	30.6	29.7	31.9	27.8
Gender	Female	75.3	79.2	77.2	77.4	71.0	87.7	77.7	76.9	80.9	75.0
	Male	24.7	20.8	22.8	22.6	29.0	12.3	22.3	23.1	19.1	25.0
Region	Midwest	20.4	22.9	18.3	28.3	17.6	28.1	21.8	20.5	20.9	35.0
	Northeast	19.4	15.1	21.8	9.4	24.4	10.5	15.6	15.4	21.7	15.0
	South	41.1	42.8	39.1	43.4	37.8	43.9	39.8	48.7	34.8	30.0
	West	19.1	19.2	20.8	18.9	20.2	17.5	22.7	15.4	22.6	20.0
FRL eligibility (%)	0–25	27.8	25.4	22.8	18.9	30.6	22.8	19.9	12.8	26.5	35.0
	25-50	30.1	27.9	27.9	34.0	23.8	26.3	29.9	20.5	27.4	20.0
	50-75	26.1	27.5	28.9	30.2	25.9	31.6	31.3	46.2	24.8	25.0
	75-100	16.0	19.2	20.3	17.0	19.7	19.3	19.0	20.5	21.3	20.0
School size	Large	61.7	58.0	53.8	47.2	45.6	56.1	43.6	51.3	47.4	65.0
	Medium	27.1	28.2	32.5	35.8	36.8	28.1	32.7	23.1	36.1	30.0
	Small	11.1	13.8	13.7	17.0	17.6	15.8	23.7	25.6	16.5	5.0
Urbanity	City	27.0	27.9	23.4	18.9	29.0	33.3	26.1	20.5	27.8	25.0
	Rural	24.6	25.1	30.5	37.7	26.4	28.1	30.8	33.3	26.5	20.0
	Suburb	37.3	34.6	32.0	34.0	32.6	29.8	31.3	25.6	31.7	50.0
	Town	11.1	12.4	14.2	9.4	11.9	8.8	11.8	20.5	13.9	5.0
Level	Elementary	45.5	49.2	57.9	60.4	48.7	54.4	57.8	59.0	57.4	40.0
	Middle	19.0	17.2	19.3	18.9	23.3	22.8	15.2	23.1	19.1	20.0
	High	32.8	31.5	19.8	18.9	24.4	22.8	23.2	17.9	17.8	35.0
	Other	2.6	2.1	3.0	1.9	3.6	0.0	3.8	0.0	5.7	5.0
Minority students	0–25	32.2	33.1	32.7	39.6	36.8	35.7	35.6	43.6	33.5	30.0
(%)	25-75	42.9	39.0	37.8	41.5	38.4	41.1	38.9	41.0	37.9	45.0
	75-100	24.9	27.9	29.6	18.9	24.7	23.2	25.5	15.4	28.6	25.0

*Note.* FRL = free/reduced price lunch.

To quantify the statistical significance of discrepancies observed, we report (for each strategy and each characteristic) a *p* value of an omnibus test that assesses (jointly across all categories of a variable) the presence of differences in the categorical frequencies of panel members versus nonresponders. These comparisons are performed using Fisher’s exact test, the results of which are shown in Table 4. For each strategy, we report an analogous *p* value that compares enrolled panel members sampled using each strategy to enrolled panelists sampled using our preferred strategy (US$10 gift card as pre-incentive). For strategies that involve refusal conversion, categorical frequencies are calculated using only educators contacted during the specific conversion effort—frequencies calculated cumulatively across phases are not reported.

**Table 4:** *p* values From Omnibus Tests to See If There Are Differences Between Responders and Nonresponders (Test 1) and Tests to See If the Differences Vary by Strategy Used (Test 2).

Test	Variable	Group 1	Group 2			Group 1a
		Promised incentive	US$10 gift card	US$20 gift card	Phone-based	Refusal conversion
Test 1: *p* values for differences between responders and nonresponders	Subject	.084	.110	.956	.683	.167
	Gender	.021	.000	.009	1.000	.557
	Region	.031	.118	.067	.701	.597
	FRL	.077	.806	.658	.278	.829
	School size	.060	.651	.362	.482	.270
	Urbanity	.478	.648	.863	.456	.419
	Level	.264	1.000	.604	.451	.240
	Minority students (%)	.096	.273	.943	.346	.875
Test 2: *p* values for differences between responders from the US$10 gift card strategy and responders from the respective strategy	Subject	.043	—	.065	.958	.048
	Gender	.728	—	.208	1.000	1.000
	Region	.656	—	1.000	.693	.723
	FRL	.640	—	.862	.311	.454
	School size	.268	—	.595	.356	.311
	Urbanity	.204	—	.372	.453	.412
	Level	.221	—	.763	.950	.281
	Minority students (%)	.333	—	.853	.960	.791

*Note.* FRL = free/reduced price lunch.

There is evidence of demographic discrepancies between enrolled and nonresponding teachers from Group 1. For example, during this phase, teachers who enrolled are more likely (than those who did not) to be females, less likely to come from the Northeast, and more likely to come from smaller schools. However, these differences are fairly minor; statistical significance is likely a consequence of the large number of teachers that were part of Group 1. Discrepancies between panelists and nonresponders sampled using the other strategies appear to be larger at times; however, these discrepancies are not statistically significant. Furthermore, teachers who were successfully recruited with the various strategies do not appear to be different (with respect to the observed characteristics) from teachers recruited using the preferred strategy. The only exception is that most groups yield a lower portion of general elementary teachers (as a subject) than is observed in the preferred strategy. This is likely an aberration that appears significant due to the multiple hypothesis tests being considered.

Note that if enrolled panelists are differentiated from nonresponding teachers across observable characteristics (such as the ones considered here), survey weighting can be used to adjust for any resulting nonresponse bias. Therefore, differentiation across unobservable domains is the primary concern. As discrepancies across observable characteristics between successfully recruited educators and refusing educators are minimal, we feel that any (recruitment-based) nonresponse bias that remains following statistical adjustments across observable predictors of noncompliance will also be minimal. Furthermore, we feel that this observation holds regardless of the recruitment strategy used.

### Survey Follow-Up

Finally, we consider the possibility that the various modes of recruitment yield differing values of completion on surveys administered to the ATP. (Recall a panel member is considered to be successfully recruited if they sign in to our web platform to register and complete a brief information form; surveys are administered to panel members at a later point, which makes it possible for a teacher to enroll in the ATP but not respond to surveys.) Surveys were administered to the teacher ATP during February 2015, June 2015, October 2015, and February 2016. Table 5 illustrates the response rates (among recruited panel members) for these surveys.

**Table 5:** Teacher Response Rates on the Various Surveys by Recruitment Group.

	*n*	February 2015		June 2015		October 2015		February 2016	
		Response rate (%)	*p* value^a^	Response rate (%)	*p* value	Response rate (%)	*p* value	Response rate (%)	*p* value
Group 1: promised incentive	711	60.5	.2402	49.9	.3158	42.8	.5651	43.5	.7722
Group 2: US$10 gift card	53	69.2	—	57.7	—	38.5	—	40.4	—
Group 2: US$20 gift card	57	69.6	1.0000	66.1	.4296	48.2	.3366	53.6	.1831
Group 2: US$20 iCard	3	100	.5481	100	.2667	100	.0675	100	.0771
Group 2: phone-based	39	68.4	1.0000	39.5	.1347	31.6	.6561	31.6	.5071
Group 1a: refusal conversion	19	47.4	.1038	31.6	.0640	21.1	.2576	31.6	.5875
Total	882	61.8	.1902	50.7	.0150	42.1	.0606	43.3	.0839

^a^*p* values compare the response rate for the “Group 2: US$10 gift card” group to that of the group in the respective row. The *p* value in the total column is for an omnibus test to see if the rates differ across all the groups.

From Table 5, we see slight evidence that the different modes of recruitment yield different response rates. However, our preferred method (US$10 gift card as pre-incentive) consistently yields one of the higher rates of response, whereas other methods (e.g., phone-based recruitment and refusal conversion) observe notably lower response rates. Also worth noting is the fact that response rates for all panel members (regardless of how they were recruited) decrease over time.

## Conclusion

Our study reveals complications that arise in the recruitment of educators into survey panels. Although low rates of survey response have been reported in previous literature on surveys of educators (20%-30% without pre-incentive), the rates of successful recruitment into the ATP reported here were even lower (14% successful recruitment without pre-incentive)—this could be a consequence of educators not wanting to make a commitment to a long sequence of surveys, the timing of contact (the workload of teachers varies greatly during the year), or perhaps the increased burden that is been placed on teachers in recent years due to policies related to testing and accountability.

Furthermore, this study yields several interesting conclusions regarding the comparative effectiveness of techniques used to recruit teachers into survey panels. First, we saw that using pre-incentives produces a higher recruitment rate than promised incentives (29.2% vs. 13.5%)—this finding is in line with previous literature. Using statistical comparisons, we observe that, despite low overall recruitment rates for all strategies, pre-incentives were not necessarily more costly than promised incentives in terms of costs per recruit. Specifically, using promised incentives, each recruited teacher cost US$70, whereas each teacher recruited using pre-incentives cost US$78; the discrepancy between these values was not found to be statistically significant (*p* = .411).

The amount of the pre-incentive did not have a statistically significant effect on recruitment success rates, although we did not consider a wide range of pre-incentive amounts. This finding is consistent with existing literature, which finds that a marginal effect of pre-incentives is most evident when values less than US$10 are considered (James & Bolstein, 1990; Trussell & Lavrakas, 2004). Alternative strategies (one involving phone-based recruitment with promised incentives and another involving pre-incentives distributed digitally) did not prove to be more successful or cost-effective than the US$10 pre-incentive. We tested similar strategies for refusal conversion after an initial promised incentive recruitment effort that demonstrated some success at recruiting educators, although in our view, these strategies were not successful enough to warrant their costs. Specifically, a two-phased strategy involving a promised incentive followed by a US$10 pre-incentive for refusing educators as a means of refusal conversion yielded a lower cumulative rate of recruitment (23.0%) than that which was observed when a single-phased, US$10 pre-incentive strategy was used (27.2%), although this difference was not statistically significant (*p* = .274).

Furthermore, exploration into the representativeness of the empaneled educators revealed only minor discrepancies in observable descriptive characteristics between nonresponders and the enrolled panelists—this provides evidence, perhaps, that (following appropriate weighting adjustments) residual bias stemming from nonresponse during recruitment will be minimal. Finally, we saw that the responsiveness of a panel member on actual surveys was not heavily dependent on the strategy used to recruit them.

Of interest is the generalizability of our findings to other populations and perhaps to other types of survey mechanisms (e.g., cross-sectional surveys). Many of our conclusions with regard to the comparison of strategies are consistent with existing literature (e.g., Church, 1993; Edwards et al., 2002; Goldenberg et al., 2009; Scherpenzeel & Toepoel, 2012; Singer & Ye, 2013)—ergo, we feel that such findings may generalize. However, the specific response rates and cost-effectiveness that we report are likely unique to our setting and will not necessarily translate to other situations.

## Declaration of Conflicting Interests

The author(s) declared no potential conflicts of interest with respect to the research, authorship, and/or publication of this article.

## Note

1 Beginning Teacher Longitudinal Study, Overview; downloaded June 18, 2012 from http://nces.ed.gov/surveys/btls/index.asp.
